# Association between psoriasis and thyroid function: results from the Brazilian Longitudinal Study of Adults Health (ELSA-Brasil)

**DOI:** 10.20945/2359-3997000000640

**Published:** 2023-06-19

**Authors:** Vandrize Meneghini, William R. Tebar, Itamar Souza Santos, Carolina Castro Porto Silva Janovsky, Bianca de Almeida-Pititto, Paulo A. Lotufo, Alessandra C. Goulart, Isabela M. Bensenor

**Affiliations:** 1 Universidade de São Paulo Centro de Pesquisa Clínica e Epidemiológica São Paulo SP Brasil Centro de Pesquisa Clínica e Epidemiológica, Universidade de São Paulo, São Paulo, SP, Brasil; 2 Universidade de São Paulo Departamento de Medicina Interna São Paulo SP Brasil Departamento de Medicina Interna, Universidade de São Paulo, São Paulo, SP, Brasil; 3 Universidade Federal de São Paulo Departamento de Medicina Preventiva São Paulo SP Brasil Departamento de Medicina Preventiva, Universidade Federal de São Paulo, São Paulo, SP, Brasil

**Keywords:** Psoriasis, triiodothyronine, thyroid-stimulating hormone, thyroxine

## Abstract

**Objective:**

To determine the relationship between psoriasis, thyroid-stimulating hormone (TSH), free thyroxine (FT4), free triodothyronine (FT3), thyroid peroxidase antibodies (TPOAb), and subclinical thyroid dysfunctions in middle-aged and older adults.

**Materials and methods:**

Cross-sectional analyses included a self-reported medical diagnosis of psoriasis and thyroid function from the 3^rd^ visit (2017-2019) of the Brazilian Longitudinal Study of Adult Health (ELSA-Brasil). TSH, FT4, and FT3 levels were analyzed as continuous variables and quintiles, and TPOAb positivity and subclinical hypothyroidism as a yes/no variable. Logistic regression models were built as crude and adjusted by main confounders (age, sex, education level, race/ethnicity, and smoking).

**Results:**

From 9,649 participants (52.3% women; 59.2 ± 8.7 years old), the prevalence of psoriasis was 2.8% (n = 270). TSH, FT4, TPOAb positivity, and subclinical hypothyroidism were not associated with psoriasis in the main analyses. In the stratified analysis, our findings showed positive associations of the lowest (OR = 2.01; 95% CI 1.05-3.84; p = 0.036) and the highest (OR = 2.13; 95% CI 1.12-4.05; p = 0.022) quintiles of FT4 and a protective association of TPOAb positivity (OR = 0.43; 95% CI 0.19-0.98; p = 0.046) with prevalent psoriasis in women. In the logistic regression for FT3, participants in the 1^st^ quintile showed a statistically significant association with psoriasis for the whole sample (OR = 1.66; 95% CI 1.11-2.46; p = 0.013) and for men (OR = 2.25; 95% CI 1.25-4.04; p = 0.007) in the sex-stratified analysis.

**Conclusions:**

The present study showed that the association of FT4 levels with psoriasis are different according to sex, with a possible U-shaped curve in women but not in men. Although there were some associations of FT3 with psoriasis, they may be a consequence of non-thyroidal illness syndrome. Further prospective data may clarify the association of thyroid function and psoriasis.

## INTRODUCTION

Psoriasis is a chronic and inflammatory autoimmune disease characterized by skin and systemic inflammation (1). The prevalence rates of psoriasis range between 0.4% and 2.8% worldwide (2) and 1.1% and 1.5% in Brazil (3). Genetic, immune, and environmental factors influence this condition (4), and it is associated with other comorbidities, such as metabolic and cardiovascular diseases (5, 6).

The etiology and pathogenesis of psoriasis are not entirely elucidated (7). The development and progression of psoriasis are related to a dysfunction in the immune system, including the T-helper 1 cell-mediated inflammation (8, 9). Autoimmune conditions share part of their genetic background and tend to coexist, in that way; patients with psoriasis have a higher risk of other immune-related diseases, such as autoimmune thyroiditis (10–12). Psoriasis and thyroiditis have a common signaling pathway (IL-23 and Th17 cells) that may explain the associations between them (7,13,14).

Despite the pathophysiology background linking psoriasis and thyroid diseases, previous studies still reported controversial findings (10,12,15–18). A recent cross-sectional study performed on a sample of American adults has shown that having psoriasis increases the odds of having thyroid disease (15). Similarly, a cohort study showed that people with psoriasis had an increased risk for incident thyroid diseases (12). Other studies with small sample sizes also found a higher prevalence of thyroid dysfunction in patients with psoriasis (10, 16). Khan and cols. investigated the association between thyroid function, assessed through the thyroid-stimulating hormone (TSH), free thyroxine (FT4), thyroid peroxidase antibodies (TPOAb), hypo- and hyperthyroidism, and psoriasis disease in a cross-sectional and longitudinal analysis using the data of 8,214 participants from the Rotterdam Study (Netherlands) (17). They found no significant association between the variables, but there was a positive trend between TSH and prevalent psoriasis and between FT4 and incident psoriasis (not significant). Vassilatou and cols. reported no significant difference in the levels of TPOAb, TSH, FT4, total triiodothyronine (T3), and total thyroxine (T4) in patients with psoriasis and controls without psoriasis (18). The authors also showed no association between psoriasis and autoimmune thyroiditis (TPOAb positivity). In Brazil, only one study found a higher prevalence of Hashimoto's Thyroiditis in a small sample of 60 patients with psoriasis compared to 60 healthy controls (19).

Besides, no study properly reported the association between thyroid hormones, subclinical conditions, and psoriasis. Thus, the present study aimed to determine cross-sectionally whether psoriasis is associated with TSH, FT4, free triiodothyronine (FT3), TPOAb, and subclinical thyroid dysfunctions in middle-aged and older adults of the Brazilian Longitudinal Study of Adult Health (ELSA-Brasil).

## MATERIALS AND METHODS

### Study design

This cross-sectional study analyzed data from the 3^rd^ visit (2017-2019) of the study after a 9-year follow-up. Detailed information about the study design is published elsewhere (20–22). Briefly, ELSA-Brasil is a prospective cohort study that examined 15,105 civil servants (35-74 years old) from six cities: Belo Horizonte, Porto Alegre, Rio de Janeiro, São Paulo, Salvador, and Vitória. Face-to-face assessments occurred at baseline visit (2008-2010), visit 2 (2012-2014), and visit 3 (2017-2019) for follow-up. The examinations and interviews were conducted in the study research centers, following strict quality control procedures by trained staff (21, 23). The six local research ethics committees approved the ELSA-Brasil protocol, and all participants voluntarily signed an informed consent form to participate in this study (CAAE Number at Plataforma Brasil: 08109612.7.1001.0076). The ELSA-Brasil study was developed according to the Declaration of Helsinki.

### Participants

**Figure 1:**
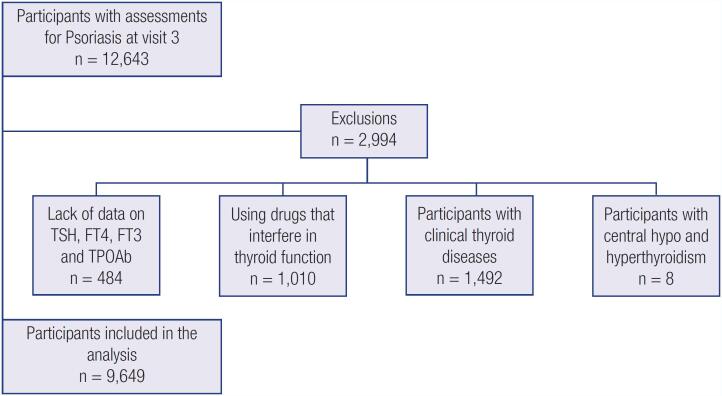
Flowchart of the participants included in the analysis.

We included in this analysis all participants with information about psoriasis and thyroid function at visit 3 (Figure 1). The main reasons for missing data on visit 3 were unavailability to come to the clinical center for examination, moving to a distant place, and death. Participants using drugs that interfere with thyroid function (amiodarone, betamethasone, biotin, carbamazepine, deflazacort, heparin, fludrocortisone, hydrocortisone, methylprednisolone, prednisolone, prednisone, oxcarbazepine, haloperidol, levodopa, lithium, metoclopramide, phenytoin, propranolol, primidone, rifampicin, valproic acid, sodium valproate, or divalproex sodium) were excluded from the analysis. In addition, we excluded participants that had overt hypo- or hyperthyroidism and with central hypo- (low free-T4 levels with inappropriately low TSH levels) or hyperthyroidism (high free-T4 levels with inappropriately high TSH levels). A total of 9,649 participants were included in this study.

### Psoriasis

The self-reported psoriasis was determined through the affirmative response to the question made in the 3^rd^ visit: “Have you been told by a physician that you had/have psoriasis?” With an affirmative answer, the next question was “What was your age when a physician for the first time told you that you have psoriasis?” Besides that, the use of the following specific medications for the treatment of psoriasis was investigated among patients with psoriasis: abatacept, betamethasone acetate, dexamethasone acetate, fludrocortisone acetate, hydrocortisone acetate, prednisolone acetate, acitretin, adalimumab, azathioprine, betamethasone, cyclosporine, quinine hydrochloride, colchicine, desoximetasone, dexamethasone, chloroquine diphosphate, beclomethasone dipropionate, betamethasone dipropionate, bumetanide, and fludroxycortide. However, after excluding participants with psoriasis using systemic steroids, only 4 participants were using other medications to treat psoriasis: acitretin (n = 2) and azathioprine (n = 2).

### Thyroid function

All participants from the 3^rd^ visit were invited to perform thyroid hormone tests. TSH (normal range: 0.4-4.0 mIU/L), FT4 (0.93-1.7 ng/dL), and FT3 (0.20-0.44 ng/dL) were determined by a third-generation immunoenzymatic assay (Roche Diagnostic, Manheim, Germany) in serum obtained from centrifuged venous blood samples taken after an overnight fast. Levels of TPOAb were measured by electrochemiluminescence (Roche Diagnostics, Mannheim, Germany) and values equal to or greater than 34.00 IU/mL were defined as positive TPOAb. Based on TSH, FT4 levels, and the use of medications to treat thyroid disorders, participants were categorized as (1) overt hyperthyroidism (low TSH, high FT4, or use of medication to treat hyperthyroidism); (2) subclinical hyperthyroidism (low TSH, normal FT4, and no use of thyroid drugs); (3) euthyroidism (TSH values within the reference range and no use of medications to treat thyroid diseases); (4) subclinical hypothyroidism (high TSH, normal FT4, and no use of thyroid drugs); and (5) overt hypothyroidism (high TSH, low FT4, or use of levothyroxine). In this analysis, we used TSH, FT4, FT3, and TPOAb from euthyroid participants or participants with subclinical thyroid conditions. TSH, FT4, and FT3 were analyzed as continuous variables and as quintiles (TSH: <1.34, 1.34-1.84, 1.84-2.38, 2.38-3.29 and ≥3.29; FT4: <1.05, 1.05-1.14, 1.14-1.22, 1.22-1.32 and ≥1.32; FT3: <0.28, 0.28-0.30, 0.30-0.32, 0.32-0.34 and ≥0.34). Subclinical hypothyroidism and TPOAb positivity were analyzed as binary variables (yes/no).

### Covariates

Sociodemographic and health-related variables were collected in the 3^rd^ visit. The variables were presented as: sex (male/female), age (years, as a continuous variable), self-reported race or ethnicity (black/mixed/white/Asian/indigenous), education (less than high school/high school and some college/complete college or more), and smoking (never/past/current).

### Statistical analysis

The normal distribution of the data was attributed to the variables with skewness and kurtosis between −1 and +1. Descriptive characteristics of the participants were presented as mean and standard deviation (SD), median and interquartile range (IQR), or absolute (n) and relative (%) distribution, according to the quintiles of TSH. Kruskal-Wallis and Chi-square (or Fisher's Exact Test) tests were applied to compare groups for continuous variables with non-normal distribution and categorical variables, respectively. Thyroid hormone levels (TSH, FT4, and FT3) did not present normal data distribution; thus, we used natural log transformation (ln). We performed logistic regression models to identify the association of prevalent psoriasis (dependent variable) with TSH, FT4, and FT3 as continuous variables and quintiles, using the 3^rd^ quintile of each variable as a reference. Logistic regression models were also performed with psoriasis and TPOAb positivity and subclinical hypothyroidism. Odds ratio (OR) and confidence intervals of 95% (CI 95%) were presented for crude and adjusted analyses (model 1: sex, age, race, education; and model 2: model 1+ smoking). The last adjusted model was repeated in a sub-analysis stratifying the sample by sex. We also performed two sensitivity analyses excluding participants with TSH > 10 mIU/L and excluding participants who reported previous cardiovascular disease (coronary heart disease and stroke), diabetes, and/or inflammatory diseases (rheumatoid arthritis, lupus erythematosus, and arthritis). SPSS for Statistics version 26.0 was used for this analysis. The level of significance adopted was p < 0.05.

## RESULTS

From a total of 9,649 participants (mean age = 59.2, ±SD = 8.7 years; 52.3% women), the prevalence of psoriasis was 2.8% (n = 270) and did not vary significantly across the quintiles of TSH (p = 0.204). There was no statistically significant difference (p = 0.217) in the prevalence of psoriasis between men (n = 140; 3.0%) and women (n = 130; 2.6%). Subclinical hyper- and hypothyroidism were detected, respectively, in one (0.4%) and 33 (12.4%) of the participants with psoriasis. Positive thyroid antibodies (TPOAb ≥ 34 UI/mL) were found in 6.3% (n = 17) of the participants with psoriasis and 8.3% (n = 776) of the participants without psoriasis. The prevalence of subclinical hypo- (p = 0.57) and hyperthyroidism (p = 1.00) and positive TPOAb (p = 0.31) showed no statistically significant difference between people with and without psoriasis.

In this current study, most of the sample was white (51.2%), completed college or more (53.5%), and never smoked (60.6%). The participants in the lowest quintile of TSH were younger (p < 0.01), had a higher proportion of women (p < 0.01), were of Black and mixed self-reported race/ethnicity (p < 0.01), smoke currently (p < 0.01), completed high school and some college (p < 0.01), and had higher levels of FT4 (p < 0.01) when compared to the highest quintile of TSH. The prevalence of TPOAb positivity was higher in the 5th quintile of TSH when compared to the others (p < 0.01; Table 1).

**Table 1:** Characteristics of the sample according to quintiles of TSH

		Total	TSH					p-value
			First quintile (<1.34 mIU/L)	Second quintile (1.34-1.84 mIU/L)	Third quintile (1.84-2.38 mIU/L)	Fourth quintile (2.38-3.29 mIU/L)	Fifth quintile (≥3.29 mIU/L)	
N		9649	1892	1923	1936	1946	1952	
Psoriasis, yes (%)		270 (2.8%)	40 (2.1%)	50 (2.6%)	60 (3.1%)	64 (3.3%)	56 (2.9%)	0.204
Age (years-old), median (IQR)		58.0 (53.0-65.0)	58.0 (52.0-65.0)	57.0 (52.0-64.0)	58.0 (52.0-64.0)	58.5 (53.0-66.0)	60.0 (54.0-67.0)	**<0.001**
FT4 (ng/dL), median (IQR)		1.18 (1.08-1.29)	1.20 (1.10-1.32)	1.19 (1.09-1.31)	1.18 (1.08-1.29)	1.17 (1.07-1.29)	1.15 (1.05-1.26)	**<0.001**
FT3 (ng/dL), median (IQR)		0.31 (0.28-0.33)	0.31 (0.28-0.33)	0.31 (0.28-0.33)	0.31 (0.28-0.33)	0.31 (0.28-0.33)	0.31 (0.28-0.33)	0.914
TPOAb positivity, n (%)								**<0.001**
	No	8856 (91,8%)	1780 (94,1%)	1795 (93,3%)	1807 (93,3%)	1790 (92,0%)	1684 (86,3%)	
	Yes	793 (8,2%)	112 (5,9%)	128 (6,7%)	129 (6,7%0	156 (8,0%)	268 (13,7%)	
Sex, n (%)								**0.008**
	Men	4605 (47.7%)	838 (44.3%)	931 (48.4%)	930 (48.0%)	928 (47.7%)	978 (50.1%)	
	Women	5044 (52.3%)	1054 (55.7%)	992 (51.6%)	1006 (52.0%)	1018 (52.3%)	974 (49.9%)	
Race/ethnicity, n (%)								**<0.001**
	Black	1526 (16.0%)	424 (22.8%)	318 (16.7%)	324 (17.0%)	250 (13.0%)	210 (10.9%)	
	Mixed	2774 (29.1%)	581 (31.3%)	583 (30.7%)	554 (29.0%)	534 (27.7%)	522 (27.0%)	
	White	4883 (51.2%)	798 (43.0%)	925 (48.7%)	959 (50.2%)	1071 (55.5%)	1130 (58.5%)	
	Asian	256 (2.7%)	41 (2.2%)	50 (2.6%)	53 (2.8%)	60 (3.1%)	52 (2.7%)	
	Indigenous	90 (0.9%)	13 (0.7%)	24 (1.3%)	20 (1.0%)	14 (0.7%)	19 (1.0%)	
Education, n (%)								**0.001**
	Less than high-school	1059 (11.0%)	193 (10.2%)	201 (10.5%)	218 (11.3%)	207 (10.6%)	240 (12.3%)	
	High-school and some college	3423 (35.5%)	746 (39.4%)	713 (37.1%)	660 (34.1%)	658 (33.8%)	646 (33.1%)	
	Complete college or more	5167 (53.5%)	953 (50.4%)	1009 (52.5%)	1058 (54.6%)	1081 (55.5%)	1066 (54.6%)	
Smoking, n (%)								**<0.001**
	Never	5826 (60.6%)	1083 (57.5%)	1153 (60.2%)	1189 (61.7%)	1207 (62.1%)	1194 (61.3%)	
	Past	2879 (29.9%)	522 (27.7%)	574 (30.0%)	561 (29.1%)	603 (31.0%)	619 (31.8%)	
	Current	912 (9.5%)	280 (14.9%)	187 (9.8%)	178 (9.2%)	133 (6.8%)	134 (6.9%)	

a Chi-square test;

b Kruskal Wallis test

In Table 2, we present the association of TSH, FT4, FT3 quintiles, TPOAb positivity, and subclinical hypothyroidism with the prevalent psoriasis disease. The quintiles of TSH and FT4, compared with the reference (3^rd^ quintile), were not associated with psoriasis (p > 0.05). When compared to the 3^rd^ quintile, the 1^st^ quintile of FT3 was associated with prevalent psoriasis in the crude analysis (OR = 1.61; 95% CI 1.09-2.38; p = 0.016) and after adjustment for sex, age, education, race (OR = 1.65; 95% CI 1.11-2.46; p = 0.013), and smoking (OR = 1.66; 95% CI 1.11-2.46; p = 0.013). The positive TPOAb (OR = 0.73; 95% CI 0.44-1.23; p = 0.236) and the subclinical hypothyroidism (OR = 1.12; 95% CI 0.77-1.63; p = 0.554) did not show a statistically significant association with psoriasis.

**Table 2:** Crude and adjusted logistic regression between psoriasis and thyroid function variables (n = 9649)

		Crude	Model 1	Model 2
TSH				
	lnTSH, per unit (mIU/L)	1.27 (1.02-1.58)*	1.22 (0.97-1.52)	1.24 (0.99-1.56)
	1^st^ quintile	0.68 (0.45-1.01)	0.71 (0.47-1.07)	0.69 (0.46-1.04)
	2^nd^ quintile	0.84 (0.57-1.22)	0.86 (0.59-1.26)	0.85 (0.58-1.25)
	3^rd^ quintile	Ref	Ref	Ref
	4^th^ quintile	1.06 (0.74-1.52)	1.04 (0.72-1.49)	1.04 (0.73-1.50)
	5^th^ quintile	0.92 (0.64-1.34)	0.90 (0.62-1.31)	0.91 (0.63-1.33)
FT4				
	lnFT4, per unit (ng/dL)	1.12 (0.47-2.67)	1.00 (0.42-2.41)	0.99 (0.41-2.37)
	1^st^ quintile	1.19 (0.78-1.79)	1.19 (0.79-1.80)	1.17 (0.77-1.77)
	2^nd^ quintile	1.32 (0.89-1.96)	1.32 (0.89-1.96)	1.31 (0.88-1.94)
	3^rd^ quintile	Ref	Ref	Ref
	4^th^ quintile	1.06 (0.70-1.60)	1.01 (0.66-1.53)	1.00 (0.65-1.51)
	5^th^ quintile	1.35 (0.91-2.00)	1.29 (0.87-1.92)	1.27 (0.86-1.88)
FT3				
	lnFT3, per unit (ng/dL)	0.55 (0.21-1.45)	0.60 (0.22-1.69)	0.58 (0.21-1.62)
	1^st^ quintile	1.61 (1.09-2.38)*	1.65 (1.11-2.46)*	1.66 (1.11-2.46)*
	2^nd^ quintile	1.46 (0.99-2.15)	1.47 (0.99-2.18)	1.47 (0.99-2.17)
	3^rd^ quintile	Ref	Ref	Ref
	4^th^ quintile	0.98 (0.64-1.50)	1.00 (0.65-1.54)	1.00 (0.65-1.53)
	5^th^ quintile	1.40 (0.96-2.04)	1.48 (1.01-2.17)*	1.46 (0.99-2.14)
TPOAb				
	Negative	Ref	Ref	Ref
	Positive (≥34 IU/mL)	0.75 (0.45-1.22)	0.73 (0.44-1.22)	0.73 (0.44-1.23)
Subclinical hypothyroidism				
	No	Ref	Ref	Ref
	Yes	1.11 (0.77-1.61)	1.10 (0.76-1.60)	1.12 (0.77-1.63)

* p < 0.05;

** p < 0.01.

Ref: reference category; ln: natural log transformed; TSH: thyroid-stimulating hormone; FT4: free thyroxine; FT3: free triiodothyronine; TPOAb: thyroid peroxidase antibodies. Adjustments: Model 1: sex, age, race/ethnicity, education; Model 2: model 1+ smoking.

For the continuous variables, natural log-transformed (ln) TSH was positively associated to psoriasis in the crude analysis (OR = 1.27; 95% CI 1.02-1.58; p = 0.035) but lost significance in adjusted models. LnFT4 (OR = 0.99; 95% CI 0.41-2.37; p = 0.977) and lnFT3 (OR = 0.58; 95% CI 0.21-1.62; p = 0.301) were not significantly associated to prevalent psoriasis.

### Sensitivity analysis

In the final regression model, stratification by sex revealed a significant and positive association of the first (OR = 2.01; 95% CI 1.05-3.84; p = 0.036) and the highest (OR = 2.13; 95% CI 1.12-4.05; p = 0.022) quintiles of FT4 in women but not in men. The first (OR = 2.25; 95% CI 1.25-4.04; p = 0.007) and second (OR = 1.82; 95% CI 1.05-3.17; p = 0.034) quintiles of FT3 were associated with psoriasis in men. Moreover, there was a negative association of TPOAb positivity with prevalent psoriasis (OR = 0.43; 95% CI 0.19-0.98; p = 0.046) for the women group (Table 3).

**Table 3:** Adjusted logistic regression between psoriasis and thyroid function variables, according to sex

		Male	Female
TSH			
	lnTSH, per unit (mIU/L)	1.27 (0.93-1.75)	1.23 (0.89-1.70)
	1^st^ quintile	0.62 (0.34-1.12)	0.73 (0.41-1.31)
	2^nd^ quintile	0.83 (0.49-1.40)	0.88 (0.51-1.54)
	3^rd^ quintile	Ref	Ref
	4^th^ quintile	1.04 (0.63-1.70)	1.07 (0.63-1.82)
	5^th^ quintile	0.83 (0.50-1.39)	0.99 (0.58-1.71)
FT4			
	lnFT4, per unit (ng/dL)	0.84 (0.25-2.79)	1.08 (0.30-3.88)
	1^st^ quintile	0.78 (0.44-1.37)	2.01 (1.05-3.84)*
	2^nd^ quintile	1.03 (0.62-1.71)	1.89 (1.00-3.60)
	3^rd^ quintile	Ref	Ref
	4^th^ quintile	0.60 (0.33-1.06)	1.82 (0.95-3.51)
	5^th^ quintile	0.86 (0.52-1.43)	2.13 (1.12-4.05)*
FT3			
	lnFT3, per unit (ng/dL)	0.28 (0.06-1.20)	1.14 (0.27-4.83)
	1^st^ quintile	2.25 (1.25-4.04)*	1.38 (0.81-2.35)
	2^nd^ quintile	1.82 (1.05-3.17)*	1.18 (0.67-2.06)
	3^rd^ quintile	Ref	Ref
	4^th^ quintile	1.10 (0.61-1.98)	0.89 (0.47-1.70)
	5^th^ quintile	1.39 (0.82-2.37)	1.67 (0.96-2.90)
TPOAb			
	Negative	Ref	Ref
	Positive (≥34 IU/mL)	1.26 (0.65-2.43)	0.43 (0.19-0.98)*
Subclinical hypothyroidism			
	No	Ref	Ref
	Yes	1.18 (0.72-1.94)	1.05 (0.60-1.85)

* p < 0.05.

Ref: reference category; TSH: thyroid-stimulating hormone; FT4: free thyroxine; FT3: free triiodothyronine; TPOAb: thyroid peroxidase antibodies. Data presented refers to Model 2 adjusted for age, race/ethnicity, education, and smoking.

Additional regression analyses were performed excluding participants who reported previous cardiovascular disease, diabetes, and/or inflammatory diseases, resulting in 201 participants with psoriasis (n = 7,608). There were positive and significant associations with lnTSH (OR = 1.40; 95% CI 1.07-1.83; p = 0.013), the 1^st^ (OR = 1.71; 95% CI 1.07-2.73; p = 0.024), and 2^nd^ (OR = 1.60; 95% CI 1.02-2.51; p = 0.041) quintiles of FT3 with psoriasis. The association of low FT3 levels with psoriasis remained positive after excluding cardiovascular diseases, diabetes, and inflammatory diseases from the sample. Detailed information is presented in Table 4.

**Table 4:** Crude and adjusted logistic regression between psoriasis and thyroid function variables, after exclusion of patients reporting cardiovascular disease, diabetes and inflammatory diseases at baseline (n = 7608; psoriasis n = 201)

		Crude	Model 2
TSH			
	lnTSH, per unit (mIU/L)	1.42 (1.09-1.84)*	1.40 (1.07-1.83)*
	1^st^ quintile	0.63 (0.39-1.01)	0.62 (0.38-1.00)*
	2^nd^ quintile	0.82 (0.53-1.28)	0.82 (0.53-1.27)
	3^rd^ quintile	Ref	Ref
	4^th^ quintile	0.94 (0.62-1.43)	0.90 (0.59-1.38)
	5^th^ quintile	1.06 (0.70-1.60)	1.03 (0.68-1.56)
FT4			
	lnFT4, per unit (ng/dL)	1.36 (0.49-3.77)	1.19 (0.43-3.29)
	1^st^ quintile	1.20 (0.76-1.90)	1.18 (0.74-1.87)
	2^nd^ quintile	1.13 (0.72-1.78)	1.11 (0.70-1.75)
	3^rd^ quintile	Ref	Ref
	4^th^ quintile	0.97 (0.60-1.56)	0.88 (0.54-1.43)
	5^th^ quintile	1.39 (0.89-2.17)	1.30 (0.83-2.04)
FT3			
	lnFT3, per unit (ng/dL)	0.46 (0.15-1.45)	0.44 (0.13-1.50)
	1^st^ quintile	1.64 (1.04-2.58)*	1.71 (1.07-2.73)*
	2^nd^ quintile	1.56 (1.00-2.43)	1.60 (1.02-2.51)*
	3^rd^ quintile	Ref	Ref
	4^th^ quintile	0.92 (0.56-1.51)	0.92 (0.56-1.52)
	5^th^ quintile	1.37 (0.89-2.11)	1.40 (0.90-2.18)
TPOAb			
	Negative	Ref	Ref
	Positive (≥ 34 IU/mL)	0.69 (0.39-1.25)	0.72 (0.40-1.30)
Subclinical hypothyroidism			
	No	Ref	Ref
	Yes	1.37 (0.91-2.05)	1.36 (0.90-2.06)

* p < 0.05;

** p < 0.01.

Ref: reference category; ln: natural log transformed; TSH: thyroid-stimulating hormone; FT4: free thyroxine; FT3: free triiodothyronine; TPOAb: thyroid peroxidase antibodies. Model 2 was adjusted for sex, age, race/ethnicity, education, and smoking.

Analyses excluding those with TSH > 10 mIU/L did not change the direction of the association, although the association of psoriasis with natural log-transformed TSH in the final adjusted model became statistically significant (OR = 1.27, 95% CI 1.01-1.59; p = 0.043; in Table 5).

**Table 5:** Crude and adjusted logistic regression between psoriasis and thyroid function variables, excluding participants with TSH > 10 mIU/L (n = 9630)

		Crude	Model 2
TSH			
	lnTSH, per unit (mIU/L)	1.29 (1.03-1.61)*	1.27 (1.01-1.59)*
	1^st^ quintile	0.68 (0.45-1.01)	0.69 (0.46-1.04)
	2^nd^ quintile	0.84 (0.57-1.22)	0.85 (0.58-1.25)
	3^rd^ quintile	Ref	Ref
	4^th^ quintile	1.06 (0.74-1.52)	1.04 (0.73-1.50)
	5^th^ quintile	0.93 (0.65-1.35)	0.92 (0.63-1.34)
FT4			
	lnFT4, per unit (ng/dL)	1.12 (0.47-2.66)	0.98 (0.41-2.35)
	1^st^ quintile	1.19 (0.79-1.80)	1.18 (0.78-1.78)
	2^nd^ quintile	1.32 (0.89-1.96)	1.31 (0.88-1.94)
	3^rd^ quintile	Ref	Ref
	4^th^ quintile	1.06 (0.70-1.60)	1.00 (0.65-1.51)
	5^th^ quintile	1.35 (0.91-1.99)	1.27 (0.85-1.88)
FT3			
	lnFT3, per unit (ng/dL)	0.55 (0.21-1.45)	0.58 (0.21-1.62)
	1^st^ quintile	1.61 (1.09-2.38)*	1.65 (1.11-2.46)*
	2^nd^ quintile	1.46 (0.99-2.15)	1.47 (0.99-2.17)
	3^rd^ quintile	Ref	Ref
	4^th^ quintile	0.98 (0.64-1.50)	1.00 (0.65-1.53)
	5^th^ quintile	1.40 (0.96-2.03)	1.46 (0.99-2.14)
TPOAb			
	Negative	Ref	Ref
	Positive (≥34 IU/mL)	0.75 (0.46-1.23)	0.74 (0.44-1.23)
Subclinical hypothyroidism			
	No	Ref	Ref
	Yes	1.13 (0.78-1.64)	1.14 (0.78-1.66)

* p < 0.05;

** p < 0.01.

Ref: reference category; ln: natural log transformed; TSH: thyroid-stimulating hormone; FT4: free thyroxine; FT3: free triiodothyronine; TPOAb: thyroid peroxidase antibodies. Model 2 was adjusted for sex, age, race/ethnicity, education, and smoking.

## DISCUSSION

This study aimed to investigate the association of psoriasis with quintiles and levels of TSH, FT4, FT3, TPOAb positivity, and subclinical thyroid dysfunction in middle-aged and older participants in the ELSA-Brasil cohort. TSH, FT4, TPOAb positivity, and subclinical hypothyroidism were not associated with psoriasis in the main analyses. However, in the stratified results according to sex, women showed a U-shaped curve association between FT4 and psoriasis, and a negative association of TPOAb positivity and psoriasis. In this sample, FT3 showed a trend for a U-shaped curve with lower and higher levels of FT3 associated with prevalent psoriasis, using the third quintile as the reference. Although the association of low levels of free-T3 remained significant after the exclusion of most common diseases in the sample, excluding an effect of other non-thyroidal diseases over thyroid function is not possible in this analysis.

Although our findings did not show an association of FT4 and psoriasis combining both sexes, it showed a positive association of the lowest and the highest quintiles of FT4 with psoriasis for the women subgroup in the stratified analyses. Gul and cols. reported higher FT4 levels in patients with psoriasis compared to controls (24), although it was not correlated to the sex. On the other hand, Arican and cols. (25), Vassilatou and cols. (18), and Khan and cols. (17) found no significant association of psoriasis with FT4 levels. Except for the study of Khan and cols. that included a large sample (17), all other studies have a small sample size making it difficult to carry out analyses stratified by sex. No studies were found regarding a relationship between FT4 and psoriasis specifically for women. Therefore, further investigations are needed to better understand this relationship. Besides that, studies usually use the comparison between lower and higher levels of thyroid hormones, so having the third quintile as the reference for these analyses could make possible another point of view regarding the relationship of FT4, FT3, and psoriasis.

We found that different quintiles of TSH were not associated with psoriasis when compared to people without psoriasis. A trend for a positive association was found for log transformed data, meaning that participants with higher levels of TSH had higher odds of having psoriasis. After excluding participants who reported previous chronic diseases, this association became statistically significant. The results from the Rotterdam Study, a cross-sectional analysis of 8,214 participants (mean age = 62.3 years old), showed no significant association between TSH and psoriasis (17). Both studies reported an association of TSH and TPOAb levels with psoriasis and included more severe cases of psoriatic arthritis (10, 26). Antonelli and cols. (10) compared 36 patients with psoriatic arthritis with 180 controls for thyroid status (TSH, FT4, FT3, and TPOAb). They showed no difference in FT3 and FT4 but significantly higher values of TSH and TPOAb for patients with psoriatic arthritis (10). In the study of Fallahi and cols. (26), participants with psoriatic arthritis (n = 97, age = 56 ± 12 years old, 47.4% women) were compared to controls from the general population with a similar age and proportion of women for thyroid status. Except for them, no other studies reported an association of TSH with psoriasis.

We found that people who had lower FT3 levels had a higher odds ratio of having psoriasis, compared to the third quintile (FT3 = 0.30-0.32 ng/dL). Arican and cols. (25) reported higher values of FT3 in patients with psoriasis. They investigated the serum levels of total T3, FT3, total T4, FT4, and TSH in 103 patients with psoriasis (66 females; mean = 38.0 ± 21.4 years old) and 96 controls. Only FT3 and total T4 were significantly higher in patients with psoriasis (25). Hansen and cols. (27) reported data from the Danish General Suburban Population Study (age > 20 years old) regarding TSH, FT4, total T3, and TPOAb. They found that participants with psoriasis (n = 1,127) only had significantly higher mean values for total T3 when compared to the matched group (n = 5,635). Du and cols. (28) investigated the relationship between different psoriasis types and thyroid dysfunction in 468 patients (mean age = 48 years old, 32% women). Sixty participants with pustular psoriasis had lower levels of FT3 when compared to psoriasis vulgaris, erythrodermic, and psoriatic arthritis patients (28), which could imply that different types of psoriasis could be linked to different levels of FT3. Although this extrapolation seems reasonable, it needs caution since the ELSA-Brasil study was largely composed of an active work sample, and only 3.6% of psoriasis cases were on any kind of treatment.

Furthermore, to test the possible effect of non-thyroid diseases, we performed a sensitivity analysis excluding from the sample participants who reported prevalent cardiovascular disease, diabetes, and rheumatic diseases. Although the association of low levels of free-T3 remained significant after the exclusion of the most common diseases in the sample, it is not reasonable to exclude a possible effect of other non-thyroidal diseases in this analysis over thyroid function. One possible mechanism for the association between low levels of FT3 and psoriasis may be the non-thyroidal illness syndrome (NTIS), which could occur in chronic diseases (29). In these cases, inflammatory cytokines are implicated in the pathogenesis of NTIS, acting not only in the periphery but also centrally in the hypothalamus and pituitary (30). IL-6, for example, is responsible for the down-regulation of the type 1 deiodinase (D1) that impairs the conversion from T4 to T3, while NF-kB inhibits D1 and also decreases thyroid hormone receptors types a and B (TRa and TRB) (31–34). The discrepancies between FT3, FT4, and TSH levels may be explained by the homeostatic control of the thyroid-pituitary axis (35). Recent data has evaluated the non-linear relationship between logTSH and FT4, especially in situations of defensive response (36–38). Also, plasma FT3 levels are maintained by a “feedforward” mechanism that links TSH with deiodinase activity and the control of body conversion from T4 to T3 (39). In fact, it seems that many adaptive layers exist to protect the basic functionality of the homeostatic feedback control from various challenges (35), including chronic illnesses, and this could explain our results for the association of low free-T3 levels and psoriasis.

In the present study, there was no significant difference in the prevalence of positive TPOAb between participants with and without psoriasis. The results only showed an association of these variables for women in the stratified analyses, whereas positive TPOAb was a protective factor for psoriasis. Hansen and cols. identified that the proportion of patients with positive TPOAb and subclinical hyper- or hypothyroidism were not significantly different between participants with psoriasis and controls (27). Vassilatou and cols. found no association between psoriasis and the presence of autoimmune thyroiditis (positive TPOAb and/or Tg-Abs) (18). In addition, there was no association of positive TPOAb with the prevalent psoriatic disease in the Rotterdam Study (17). However, the two studies that included only cases of psoriatic arthritis reported an association with TPOAb (10, 26).

Regarding the results about subclinical thyroid diseases, our study showed no significant association with psoriasis. These findings differ from Namiki and cols. (40), who reported a 14.1% prevalence of thyroid dysfunction in a sample of 85 patients with psoriasis. Compared to our study, Du and cols. (28) observed a lower prevalence of subclinical hypothyroidism (2.6% *vs.* 14.3% in this study) and a higher prevalence of subclinical hyperthyroidism (5.3% *vs.* 0.8% in this study). However, only one participant presented subclinical hyperthyroidism in our analysis. Therefore, our study has no statistical power to determine this association with subclinical hyperthyroidism.

There are some mechanisms underlying the association between psoriasis and thyroid dysfunctions, and the immune predominance of Th1 chemokines is an important one. CXCL10, the Th1 prototype chemokine, can be found in keratinocytes and psoriatic plaques, and it is sensitive to effective treatment of active plaque (8, 9). CXCL10 is also increased in autoimmune thyroiditis (41). Higher serum levels of CXCL10 were found in a sample of 28 participants with psoriatic arthritis and autoimmune thyroiditis when compared to 37 participants with only psoriatic arthritis and 65 controls, suggesting a common path between both diseases (42). Another common signaling pathway between psoriasis and autoimmune thyroid is related to IL-23 and Th17 cells (7,13,14).

This study has some limitations. Participants were not clinically evaluated to analyze the severity and type of psoriasis, and the diagnosis of psoriasis was based on a self-reported previous medical diagnostic, which is considered good information since there is no biomarker related to psoriasis diagnosis (43). However, we understand that most of our cases of psoriasis are mild, considering that ELSA-Brazil is an occupational cohort comprised of 80% active workers, with low use of specific medications. Moreover, medical self-report of psoriasis is used as a diagnosis of psoriasis in some studies (15, 27). The number of cases of subclinical thyroid diseases in the sample, especially in the case of subclinical hyperthyroidism, may compromise finding a possible association in the sample. Furthermore, participants are still young with a median age lower than 60 years, and both subclinical thyroid disease and psoriasis increase in older ages. Otherwise, the strengths of this study are the data analysis of a large sample from an established cohort study, assessment of participants from six different states in Brazil with centralized training of the study team, and data collection under strict quality control. In addition, the measurement of thyroid function was very comprehensive including TSH, FT4, FT3, and TPOAb.

In summary, our results identified an association between thyroid hormone levels and prevalent psoriatic disease. In the stratified analysis, our findings showed a U-shaped curve in the association of FT4 and psoriasis in women. There was a positive association of low FT3 levels with psoriasis, especially for men. However, it is impossible to discern if these results may be a consequence of non-thyroidal illness syndrome. We found no significant association for TSH values, subclinical hypothyroidism, and psoriasis in this Brazilian cohort of middle-aged and older participants.
